# Dataset of mineral contents of fine-sand and silt-sized soil particles from deserts in China and Mongolia

**DOI:** 10.1016/j.dib.2021.106879

**Published:** 2021-02-13

**Authors:** Tong He, Jessica Gray, Ying Gu

**Affiliations:** a School of Geographic Sciences, Nanjing University of Information Science and Technology, China; b The Julius Kruttschnitt Mineral Research Center (JKMRC), Queensland University, China

**Keywords:** Single-particle minerals, Soil particles, Geochemistry, Mineral contents, Mineral grouping, In-situ mineral mapping, Automated mineralogy, Chinese deserts

## Abstract

There are over 70 types of mineral species in desert soils. Previous data have focused on major mineral contents and thus, the identification of minor mineral species is lacking. The diversity of minor mineral species was investigated in 19 surficial sediments from deserts in China and Mongolia. A modern scanning electron microscope (SEM)-based in-situ mineral mapping technique was used to determine the minor mineral species concentrations. For further analysis and interpretation, the identified species were grouped into felsic, mica, carbonate, heavy, rare earth, and salt-type minerals. The data in this article demonstrate that the concentrations of felsic, mica, and carbonate minerals are higher than those of the other mineral groups, and thus can be used to provide evidence of sediment provenance. The obtained mineral concentrations were converted from the relative area percentage for each mineral species using standard mineral density data. Mineral mapping was performed using the mineral liberation analysis platform, and on average, approximately 40,000 single particles per sample were analyzed to achieve an accurate quantification of the mineral concentrations. For each of the analyzed single particles, the particle shape parameters, such as particle length and width, were stored and can be used to trace the sediment transport process. For a deeper interpretation of the data presented herein, please see the related research article “Provenance of Fe in Chinese Deserts: Evidence from the geochemistry and mineralogy of soil particles” [1].

## Specifications Table

**Table:** 

Subject	Earth and Planetary Sciences; Earth-Surface Processes			
Specific subject area	Geochemistry, soil sciences, mineralogy			
Type of data	Table			
How data were acquired	State how the data were acquired: SEM, energy dispersive spectral (EDS). Instruments: FEI Quanta 650 SEM, Bruker QUANTAX EDS. Software: Mineral Processing Tool version 2.9., Dataview version 2.9, Imageview version 2.9			
Data format	Analyzed and processed results in excel file format			
Parameters for data collection	Modal mineral abundances including 66 mineral species for the fine-sand to silt-sized particles in the desert sediments. The studied deserts: Taklamakan, Qaidam, Badain Jaran, Mu Us, Hobq, Tengger, and Mongolian Gobi. The reported mineral groups: felsic, mica, carbonate, heavy, rare earth, and salt-type minerals. The analyzed particle size range: 5–120 *µ*m.			
Description of data collection	Modal mineral abundances were generated based on the mineral liberation analysis platform [2]. Mineral identification is based on EDS spectra matching with the standard spectral library, using the Mineral Processing Tool version 2.9. In-situ mineral mapping could provide the particle shape parameters, including particle areas for each analyzed single-particle. Mineral concentrations were converted from the relative area percentages for each mineral species using the mineral species density.			
Data source location	Region: Asia Pacific Country: China Geographic locations of sampling: Mongolian Gobi Desert surface soils were sampled along a track in the region E100°–109° and N42°–46°. The following Chinese deserts were investigated in this study: the Taklamakan and Qaidam deserts in northwestern China; and the Badain Jaran, Tengger, Mu Us and Hobq deserts in northern China. The coordinates for each of the sampling sites were listed in the following table.			

	Desert	SID	Latitude/°	Longitude/°

	Taklamakan	TK10	38.23	85.35
		TK14	37.18	82.85
		TK25	41.11	82.43
		TK30	38.61	80.97
		TK40	39.46	78.09
		TK41	39.92	78.47
	Qaidam	W01	37.90	91.84
		W04	36.03	97.78
		W06	36.46	94.37
		W07	37.36	95.49
	Badain Jaran	BJ06	42.02	101.58
		BJ09	40.93	100.63
	Mu Us	MS02	37.82	107.40
		MS11	38.79	106.73
	Hobq	BQ03	40.45	109.61
	Tengger	TGL24	38.94	103.36
	Mongolian gobi	MG05	44.48	110.03
		MG14	43.31	105.87
		MG25	44.62	102.38
Data accessibility	Submitted with article			
Related research article	for a co-submission: T. He, Y.B. Sun, J. Gray, Y. Gu, Provenance of Fe in Chinese Deserts: Evidence from the Geochemistry and Mineralogy of Soil Particles, Catena. https://doi.org/10.1016/j.catena.2020.105053			

## Value of the Data

• The dataset provides insights into the full set of mineral species in Chinese desert, which comprises the minor and major mineral species without missing trace minerals.
• The generated data provide valuable insights for provenance and weathering tools based on geochemistry and mineralogy, which are of interest in many fields, including the soil sciences, paleogeography, paleoclimate, regional geology, and petrology.
• The data are useful for the future design of soil weathering tools for soils across the semi-arid area in North China to the semi-moist area in South China.
• The datasets provide the most complete iron mineralogy in the desert soils of China. Based on the iron speciation in phases, we can accurately estimate the iron concentration in easily weathered silicate minerals that can be used as weathering tools in soil sciences.
• The data clearly show that the felsic, mica, and carbonate minerals substantially restrict the main constituents of the Chinese and Mongolian Gobi deserts, thereby presenting the constitutes of the upper continental crust in the Asian interior.
• Minor mineral species are valuable for better understanding and determining sediment provenance and sediment transport.

## 1 Data Description

**Fig. 1:**
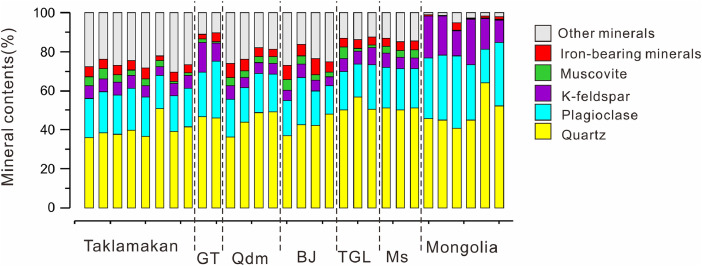
Modal mineral abundances for fine-sand to silt-sized fractions of desert sediments in East Asia. Gurbantunggut (GT), Qaidam (Qdm), Badain Jaran (BJ), Tengger (TGL), Mu Us (Ms).

The data in this article show that the single-particle mineral mapping technique can rapidly identify minor mineral species in desert soils without missing trace minerals, such as rare earth mineral species. Supplementary Table 1S summarized the concentrations of identified minor and major mineral species with respect to geographical changes from the Chinese to the Mongolian Gobi deserts. The concentration variability was quantified over seven deserts, including felsic, mica, and iron-bearing minerals. The analyzed and processed data are listed in Supplementary Table 1S. By using the modal mineral abundances in Fig. 1, the spatial variability of the mineral contents was demonstrated.

Note that the iron-bearing minerals are examined in the reference article [1]. The iron-bearing silicate mineral species are the primitive source of iron in naturally occurring soil particles generated from arid zone. The concentrations of the identified 32 species of iron-bearing silicate minerals [3] are summarized separately in Supplementary Table 2S. The iron concentrations for the mineral species are also listed in Supplementary Table 2S. Among the identified species, hornblende, chlorite, and epidote contributed most significantly (over 60%) to the iron partition for all the samples [1]. This dominance resulted from higher mineral abundances and higher iron concentrations for the three species (Supplementary Table 2S).

## 2 Experimental Design, Materials and Methods

**Fig. 2:**
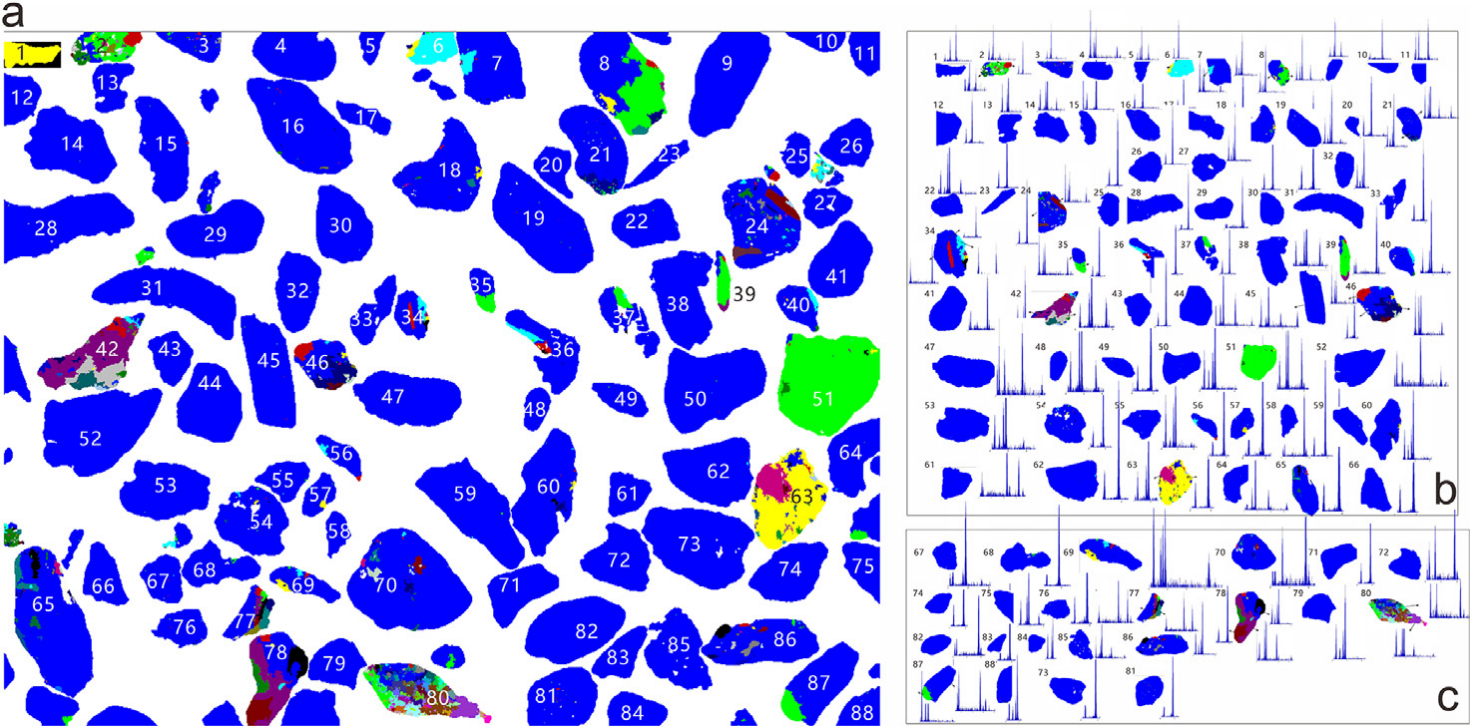
Computer-aided mineral particle segregation from polymer background (a). The stored EDS spectra for each of the single-particle (b and c).

The hardware system is composed of an FEI Quanta 650 SEM with a Bruker energy dispersive spectral (EDS) detector. The operation process was conducted using mineral liberation analysis (MLA) software, wherein the measurement was set at a magnification of 800 pixels and a frame resolution of 1024 × 800 pixels. All images were obtained under the SEM using back scattered electron (BSE) mode. For each sample, about 300 image frames were captured and each frame contains approximately 100 single-particles (Fig. 2). The computer-aided particle segregation was operated to separate each of the images for single-particles (Fig. 2a).

The high-resolution images were stored and processed by Particle X-ray Tool in the Mineral Processing Tools software (ver 2.9). The areas inside the single-particle were highlighted by colors after detailed EDS mapping pixel by pixel. That is the stored spectra and the images are correlated (Figs. 2b and 2c). The obtained spectra were stored in a separated file with ASCII format. The raw data files, named the X-ray Spectra Data were provided in the supplementary materials (e.g. BJ06, X-ray Spectra Data 1 to Data 4 in supplements). The dataset Data 1 to Data 4 in supplement which is about 600 MB in disk space was obtained from the sample BJ06.

This dataset contains 40,000 single-particles for each analyzed sample (Data 1 to Data 4). The complicated particle and petrology texture (e.g. particle number 42, 63 and 80 in Fig. 2) resulted in many more analyzing spots. Thus approximately 100,000 EDS spectra were obtained for each sample. The detailed spectrum for each spot was described by 2000 bands that is the measurement range from 0 to 200 kev with the step size of 0.1 kev. All information was presented in the row of excel files (X-ray Spectra Data 1 to Data 4).

The areas with the same EDS spectrum were assigned the same color. Until the steps above, the potential mineral phase in single-particle remains unknown. Identification of each mineral species was achieved by matching the stored EDS spectra (Figs. 2b and 2c) with the standard mineral spectra library. This library is constructed before an automated run and involves a collection of high-quality X-ray spectra for each mineral in the sample. The measurement conditions, such as beam energy (e.g., KeV), are reflected in this library, providing an elemental deportment that better reflects the chemistry of a specific set of samples. The spectra identification and matching process involve an error-based search for the measured spectrum in the standard library to find the most probable fit. Statistics on spectra pattern matching were examined to assess the confidence of classification for specific minerals.

Although testing errors for mineral identification remains, the application of the similarity probability parameter shows fundamental improvements compared with traditional SEM-EDS work. For the identification of the 32 species of iron-bearing silicate minerals, the experimental setup was similar to those aforementioned, wherein the similarity probability was used to increase the accuracy of identification for analyzing each of the single particles.

A total of 40,000 single particles were imaged and analyzed for each sample. Digital images were obtained for each of the analyzed particles. These high-resolution images were then subjected to a gray-scale histogram analysis, which allows the MLA system to discriminate the phases within each particle [2], [4]. Using X-ray in-situ mapping on the single particles, the texturally complex particles, such as perthite, were reported in the total area of K-feldspar and albite (μm^2^) separately. This mineral mapping result (for all 40,000 particles) was reported in the total area for each mineral species and then converted to a weight percentage by multiplying the mineral density. Based on this dataset, the quantitative analysis method can lower the testing errors and uncertainty present in previous methods, such as traditional SEM-EDS, optical resolution, and X-ray diffraction. In previous quantitative work, the testing error was usually larger than 10%–20% [5].

## Ethics Statement

Please refer to our Guide for Authors for more information on the ethical requirements for publication in *Data in Brief*.

## CRediT Author Statement

**Tong He**: Visualization, Investigation, Writing - Original draft preparation, Writing - Reviewing and Editing; **Jessica Gray**: Validation; **Ying Gu**: Conceptualization, Methodology, Software, Supervision.

## Declaration of Competing Interest

The authors declare no conflict of interest.

## Appendix Supplementary materials

Image, application 1

Image, application 2

Image, application 3

Image, application 4

Image, application 5

Image, application 6
